# Efficacy of a long-term home parenteral nutrition regimen containing fish oil-derived n-3 polyunsaturated fatty acids: a single-centre, randomized, double blind study

**DOI:** 10.1186/s12937-018-0419-x

**Published:** 2018-11-30

**Authors:** Helene Bohnert, Max Maurer, Philip C. Calder, Johann Pratschke, Paul Thul, Verena Müller

**Affiliations:** 1 Charité-Universitätsmedizin Berlin, Department of Surgery, Campus Charité Mitte | Campus Virchow-Klinikum, Chariteplatz 1, 10117 Berlin, Germany; 2 Human Development and Health, Faculty of Medicine, University of Southampton, Southampton, UK; 3 NIHR Southampton Biomedical Research Centre, University Hospital Southampton NHS Foundation Trust and University of Southampton, Southampton, UK

**Keywords:** Home parenteral nutrition, Lipid emulsion, N-3 polyunsaturated fatty acids, Fish oil

## Abstract

**Background:**

Data on the use of lipid emulsions containing fish-oil (FO) derived n-3 polyunsaturated fatty acids (n-3 PUFAs) in addition to medium- and long-chain triglycerides (MCT/LCT) for long-term home parenteral nutrition (HPN) are limited. This study aimed to compare HPN regimens containing either MCT/LCT/FO-derived n-3 PUFAs (test group) or MCT/LCT (control group) with respect to efficacy and safety during 8 weeks of HPN using a non-inferiority trial design with change of body mass index (BMI) as primary endpoint.

**Methods:**

This prospective, randomized, double-blind study was conducted at the Charité, Berlin, Germany, from 02/2008 until 01/2014. Adult patients (*n* = 42; aged 18 to 80 years) requiring HPN for at least 8 weeks were randomly assigned to the test or control group. Assessments included weight, height, physical examination (cardiovascular system, abdomen, respiratory tract, liver, spleen, kidney, urine tract, skin, mucous membrane, neurology, psyche, musculoskeletal system, lymph nodes), bio impedance analysis, calorimetry, blood samplings (haematology, biochemistry, fatty acid analysis) and quality of life questionnaire.

**Results:**

BMI increased in both groups with 8 weeks of HPN (ΔBMI_(test group)_ = 1.3 ± 1.1 kg/m^2^; ΔBMI_(control group)_ = 0.6 ± 0.9 kg/m^2^) demonstrating non-inferiority of the test regimen regarding nutritional efficacy. Assessment of secondary efficacy endpoints revealed that after 8 weeks of HPN with the test regimen, the proportion of n-3 PUFAs in serum, platelet and red blood cell phospholipids significantly increased, while the proportion of n-6 PUFAs decreased. The fatty acid pattern in the control group remained mostly stable. No statistically significant differences were detected between groups regarding inflammatory markers or quality of life. Laboratory parameters reflecting the safety endpoints liver function, bone metabolism, renal function, metabolic activity, lipid metabolism, coagulation and haematology were stable in both groups and no group differences were detected regarding (serious) adverse events.

**Conclusions:**

The HPN regimen prepared with MCT/LCT/FO-derived n-3 PUFAs was at least as efficient in maintaining or even improving nutritional status during HPN as the control MCT/LCT regimen. Administration of FO-derived n-3 PUFAs for 8 weeks altered the fatty acid pattern of serum, platelet and red blood cell phospholipids. Both regimens were safe and well tolerated.

**Trial registration:**

www.clinicaltrials.gov, registration number: NCT00530738.

**Electronic supplementary material:**

The online version of this article (10.1186/s12937-018-0419-x) contains supplementary material, which is available to authorized users.

## Introduction

Home parenteral nutrition (HPN) was first introduced in the early 1970s and is nowadays an established therapy in the home care setting in western countries, as morbidity and mortality associated with HPN are low [1]. HPN aims to provide adequate amounts of amino acids, glucose, lipids, electrolytes and water in order to prevent malnutrition in patients requiring long-term parenteral nutrition (PN) due to prolonged gastro-intestinal tract failure [1, 2]. As prolonged malnutrition leads to weight loss, reduction of quality of life, increase in morbidity and mortality, and is associated with poor clinical outcome due to slow wound healing or impaired immune response [3–5], HPN intends to improve the patient’s clinical prognosis and quality of life.

During the last decade it has been recognized that lipid emulsions administered as part of the PN regimen not only function as a source of energy: lipid emulsions provide physiologically active polyunsaturated fatty acids (PUFAs), namely n-6 PUFAs and n-3 PUFAs. These PUFAs are incorporated into phospholipids of serum and cellular membranes and are metabolized into bioactive mediators [6]. Mediators derived from the n-6 PUFA arachidonic acid (2-series prostaglandins and thromboxanes, 4-series leukotrienes) generally exert pro-inflammatory effects, while n-3 PUFAs are converted into far less inflammatory mediators (3-series prostaglandins and thromboxanes, 5-series leukotrienes) and even to mediators that are anti-inflammatory and inflammation resolving (e.g. resolvins, protectins, maresins) (reviewed in [6–8]). Depending on their content of n-6 PUFAs and n-3 PUFAs, lipid emulsions can thus exert influence on inflammatory and immune functions [9, 10].

Several lipid emulsions are available that differ in terms of lipid composition [11]. Lipid emulsions derived from soya-bean oil deliver long-chain triglycerides (LCT) and are rich in n-6 PUFAs (mainly linoleic acid, the precursor of arachidonic acid). Those based on soya-bean and coconut oil deliver medium- and long-chain triglycerides (MCT/LCT) and have a reduced content of n-6 PUFAs compared with soya-bean oil. Lipid emulsions based on soya-bean oil, coconut oil and oil from cold-water fish (i.e. fish oil; FO) deliver a mixture of MCT, LCT and FO-derived n-3 PUFAs and have a reduced content of n-6 PUFAs while being rich in n-3 PUFAs.

The safety of parenteral administration of FO containing lipid emulsions has been established in several clinical trials and beneficial effects of FO supplementation including modulation of inflammatory markers, reduced length of hospital stay as well as reduced infectious morbidity have been shown for surgical patients [12–19], as reviewed in [20]. Concerns have been raised regarding an increased risk of bleeding due to the administration of n-3 PUFAs, based on early observations in the Greenland Inuit population which indicated a longer bleeding time associated with high consumption of fish [21]. However, clinical trials found no evidence for an increased risk of bleeding upon n-3 PUFA administration [22, 23].

Data on efficacy and safety of FO containing regimens during long-term PN in the home-care setting are limited [1]. Indeed, a recent systematic review [24] identified only one randomised controlled trial of FO-containing HPN in adult patients which indicated that long-term administration of n-3 PUFAs in the setting of HPN was safe for a period of four weeks and led to an increased n-3/n-6 PUFA ratio in plasma and red blood cells [25].

The current clinical trial extended the study duration and for the first time a period of eight weeks of HPN with n-3 PUFAs was assessed. The trial was designed to show non-inferiority of an HPN regimen prepared with a MCT/LCT/FO-derived n-3 PUFA containing lipid emulsion as compared to a conventional HPN regimen without FO-derived n-3 PUFAs with respect to nutritional efficacy (primary endpoint: change of body mass index (BMI) after 8 weeks of HPN). Secondary endpoints of this clinical trial covered safety parameters and assessed potential beneficial effects of such a regimen on quality of life and body composition as compared to a conventional HPN regimen. The aim of this study was to provide evidence that an eight week treatment with HPN containing FO-derived n-3 PUFAs is as efficient and safe as HPN without n-3 PUFAs.

## Materials and methods

### Study design

This was a prospective randomized, double-blind, single centre Phase-IV-study with two parallel groups. It was conducted at the Department of Surgery at the Charité, Berlin, Germany, from February 2008 until January 2014 in accordance with the principles of the Declaration of Helsinki and requirements of Good Clinical Practice. The conduct of the study was approved by the German Federal Institute for Drug and Medical Devices (BfArM) and approval was provided by the Ethic Committee of Berlin (LAGeSo). Informed consent was obtained from all participating patients prior to any study procedure. This study was sponsored by B. Braun Melsungen AG, Germany. A populated CONSORT checklist is provided as Additional file 1.

### Patient population

Male and female patients aged between 18 and 80 years in need of long-term HPN for at least 8 weeks recruited from the ambulatory nutritional service at the Department of Surgery at the Charité, Berlin, Germany, were considered for study participation. Eligible patients had insufficient absorption capacity not compensable by enteral nutrition (EN), and were mentally and physically capable of adhering to study procedures. Patients with contraindications for parenteral nutrition and infusion therapy were excluded from study participation as well as patients suffering from (severe) sepsis, septic shock, autoimmune disease, hemodynamic failure of any origin, alterations of coagulation, ketoacidosis within 7 days prior to enrolment, renal insufficiency, severe liver dysfunction, lipid disorders, or necrotizing pancreatitis. Further reasons for exclusion were hypersensitivity to egg-, soya-, and fish proteins or any of the ingredients of the test or reference investigational products, pregnancy and lactation, known or suspected drug abuse and participation in another clinical trial.

### Nutritional regimen

HPN regimens were either prepared using the test lipid emulsion (MCT/LCT/FO-derived n-3 PUFAs; Lipidem® 20%) or the reference lipid emulsion (MCT/LCT; Lipofundin® MCT 20%). Test and reference lipid emulsions only differed in terms of lipid composition: the test lipid emulsion contained MCT, LCT and FO-derived n-3 PUFAs in a ratio of 5:4:1, while the reference lipid emulation contained MCT and LCT in a 1:1 ratio. The test lipid emulsion contains 3.69 ± 0.14 wt% and 2.53 ± 0.14 wt% of the n-3 PUFAs eicosapentaenoic acid and docosahexaenoic acid. [26]. For regimen preparation, test or reference lipid emulsion (bottles of 500 mL, composition see Table 1) as well as vitamins and trace elements (according to individual needs) were added to a NuTRIflex® plus 2-chamber bag (1500 ml, 1190 kcal, containing amino acids and glucose) via a transfer set. In line with the routine of the investigator, i.e. the treating physician, 44.5% of caloric intake was provided in the form of lipids. HPN-patients were trained in preparing the all-in-one admixture and administered the PN regimen as a continuous infusion overnight via a central venous catheter. Depending on individual caloric requirements (calculated based on the results of indirect calorimetry performed during the baseline visit (BL) or based on estimated needs of about 25 to 35 kcal/kg/day), PN treatment was administered on 4 to 6 nights per week in order to cover at least 70% of caloric needs. Both PN regimens contained sufficient soya-bean oil to cover essential fatty acid requirements [1]. Patients were randomly assigned to either the test group (receiving MCT/LCT/FO-derived n-3 PUFAs) or to the control group (receiving MCT/LCT) in a 1:1 ratio according to a randomisation list (chronological enrolment number corresponding to a random number) prepared by an independent statistician prior study start using a permuted block design with varying block size of 2, 4 and 6. Test and reference lipid emulsions were labelled at the manufacturing site. To assure blinding of study participants, investigators and staff involved in this study, labels displayed only the patient’s random number and the content as ‘20% lipid emulsion’. After a blind data review meeting and subsequent data base lock, data was unblinded for statistical analysis.

**Table 1:** Composition of test and reference lipid emulsion (per 500 mL)

Substance	Test lipid emulsion (Lipidem® 20%)	Reference lipid emulsion (Lipofundin® MCT 20%)
MCT	50.0 g	50.0 g
LCT (soybean oil)	40.0 g	50.0 g
Fractionated FO	10.0 g	0.0 g
Egg yolk phospholipids	6.0 g	6.0 g
Glycerol	12.5 g	12.5 g
Essential fatty acids		
• Linoleic acid (n-6)	19.2–23.2 g	24.0–29.0 g
• α-Linolenic acid (n-3)	2.0–4.4 g	2.5–5.5 g
• Eicosapentaenoic acid +Docosahexaenoic acid (n-3)	4.3–8.6 g	0.0 g
Energy content	3995 kJ (955 kcal)	3995 kJ (955 kcal)
Osmolality	~ 410 mOsm/l	~ 380 mOsm/l
Titration acidity/−alkalinity (pH 7.4)	< 0.5 mmol/l	< 0.5 mmol/l
pH-value	6.5–8.5	6.5–8.5

### Investigations

Study related investigations were performed during a baseline visit (Day 1, BL), 4 weeks ±5 days (V1) and 8 weeks ±5 days (V2) after study start. BMI, used to calculate the primary endpoint ΔBMI, was determined at BL, V1 and V2 based on patient’s weight (weighing in underwear with the same scale at all time points) and patient’s height (measured according to clinical routine) using the formula: BMI = (weight [kg])/(height [m])^2^. In addition, the following secondary efficacy endpoints were determined at BL, V1 and V2: bio impedance analysis (BIA, equipment: Data Input GmbH), fatty acid pattern in erythrocytes, platelets and serum phospholipids, markers for inflammatory state (IL-6, IL-10, TNF-alpha, CRP), and quality of life questionnaire (EORTC QLQ-C-30, Version 3.0; scores calculated according to EORTC scoring guidelines). For organizational reasons (no successor found for the person in charge of blood sample processing on the ward), fatty acid pattern was assessed for a subset of patients only.

Routine laboratory parameters were determined at BL, V1 and V2 to assess the following safety endpoints: liver function (Alanine Transaminase, Aspartate Transaminase, γ-Glutamyl-Transferase, Alkaline Phosphatase and Bilirubin), bone metabolism (Ostase), renal function (Creatinine and Urea), metabolic activity (Albumin, Lactate, Glucose, pH, Sodium, Potassium, Calcium, Magnesium and Chloride), lipid metabolism (Triglycerides, Total cholesterol, HDL cholesterol, LDL cholesterol, Vitamin E), coagulation (activated Partial Thromboplastin Time, Prothrombin Time, platelet count) and haematology (Leukocyte count, Erythrocyte count, Haematocrit, Haemoglobin, Transferrin). Blood samples were collected and processed according to routine procedures at the Charité laboratory. (Serious) adverse events ((S)AEs) were recorded continuously throughout the study.

Furthermore, physical examination of cardiovascular system, abdomen, respiratory tract, liver and spleen, kidney and urine tract, skin and mucous membrane, neurology and psyche, musculoskeletal system and lymph nodes was performed, and vital signs were assessed.

All investigations except fatty acid pattern were part of the routine assessment at the trial site. Fatty acid patterns were determined at the Faculty of Medicine, University of Southampton, according to established methods of fatty acid extraction, fatty acid methyl ester (FAME) formation and FAME separation using gas chromatography (for details see [27, 28]). FAME were detected by flame ionization detection and identified by comparison with run times of authentic standards. Peak areas and the percentage contribution of each peak to the total were calculated.

### Statistics

This study was designed to show non-inferiority of the test lipid emulsion regarding the primary endpoint ‘difference of BMI between V2 and BL’ (ΔBMI). The non-inferiority margin for the treatment difference ΔBMI_test_ – ΔBMI_control_ was defined as − 1.1 kg/m^2^ based on the following assumptions:

- For patients considered for study participation, cachexia is one of the most life threatening risks and maintenance of BMI (ΔBMI_test_ = 0) is a clinical success.
- Based on routinely generated data in everyday practice at the study site, BMI increase over 8 weeks HPN support in adults was expected to be 1.1 kg/m^2^ (i.e. ΔBMI_control_ = 1.1 kg/m^2^).

Sample size was initially calculated based on routinely generated data in everyday practice at the study site indicating an average BMI increase over 8 weeks of 1.1 kg/m^2^ with a standard deviation σ of 1.57 kg/m^2^ (significance level α = 0.025, power 1-β = 0.80). Considering a drop out rate of 10%, sample size was determined to be 74, i.e. 37 patients per group. In the course of this study, the standard deviation of BMI increase was adjusted to σ = 1.07 kg/m^2^ based on the evaluation of data originating from the pilot phase [29], and sample size calculation was amended accordingly resulting in 32 completely evaluable subjects. Considering a drop out rate of 30% as experienced in the pilot phase, sample size was determined to be 42, i.e. 21 patients per group. As the Per Protocol population (PP) is the more conservative patient population in non-inferiority trials, the primary endpoint, ΔBMI, was assessed by PP (i.e. all eligible patients without major protocol violations) with a one-sided 97.5% confidence interval (CI) using a parametric one-sided t-test. Non-inferiority was postulated if the lower bound of this confidence interval was above − 1.1 kg/m^2^. In addition, analysis of the primary endpoint in the Full-analysis-set (FAS), comprising all patients who got the treatment at least once and a statement regarding the primary endpoint is possible, was planned. In this study, PP and FAS comprised the same patients and PP and FAS were therefore identical.

Demographic and anamnestic parameters were analysed in the Intent-to-treat population (ITT, all patients who received study medication at least once) and in the PP population. Statistical analyses of secondary efficacy parameters were performed by PP and FAS. Safety parameters were assessed in the ITT population only. Depending on the type of variable, Mann-Whitney or Kruskal-Wallis test (ordered categorical counts, non-paired data), Wilcoxon or Friedman test (ordered categorical counts, paired data), exact Fisher test (dichotomous variables), Pearson chi-square test (categorical character with more than two categories), or McNemar test (paired categorical data) were used. Data are presented as mean ± standard deviation (SD). Means were compared via the 2-sample t-test or analysis of variance (ANOVA) as appropriate and the significance level was defined as 5%.

## Results

### Study population

**Fig. 1:**
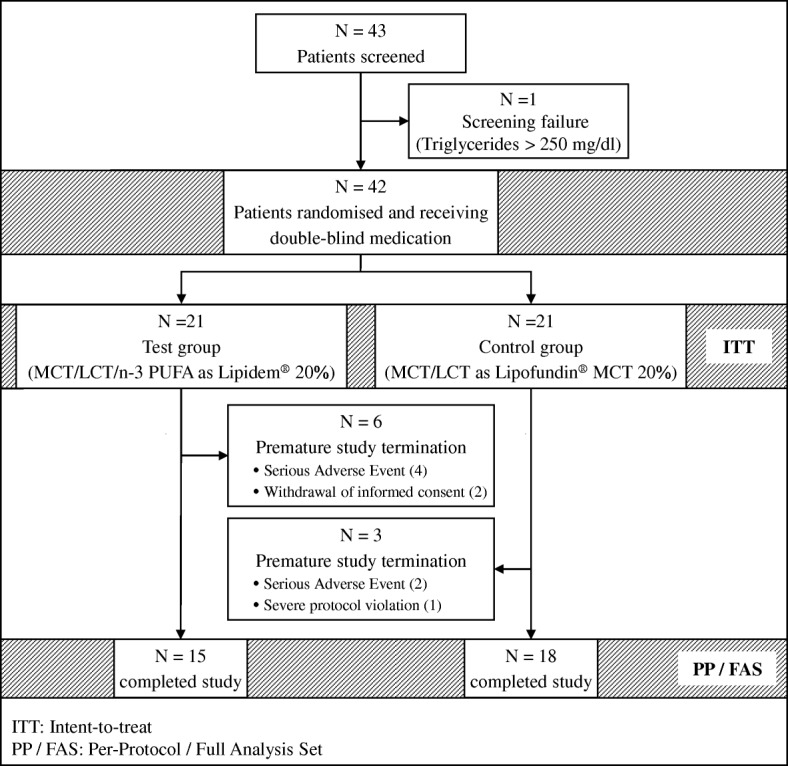
Flowchart of study patients. Figure displays the number of patients screened, randomised and included for ITT and PP / FAS analyses. Nine patients of the ITT population were excluded from PP analysis because of premature study termination due to severe protocol deviation (*N* = 1), withdrawal of informed consent (*N* = 2) or serious adverse events (SAEs; *N* = 6). SAEs leading to premature study discontinuation were not investigational product related but required discontinuation of study medication due to necessary hospitalisation

A total of 43 adult patients requiring HPN for at least 8 weeks, recruited from the ambulatory nutritional service at the University Hospital of the Charité Berlin, were screened for study participation. 42 patients were eligible and randomly assigned to receive either the test lipid emulsion (MCT/LCT/FO-derived n-3 PUFAs as Lipidem®, test group) or the reference lipid emulsion (MCT/LCT as Lipofundin®, control group). A total of nine patients prematurely discontinued the study (*n* = 6 and *n* = 3 in test and control groups, respectively, see Fig. 1 for further details). ITT analyses comprised data of 42 patients (*n* = 21 in each treatment group) while PP analyses were based on data from 33 patients (*n* = 15 and *n* = 18 in the test and control group, respectively). FAS and PP were identical in this study.

Test and control group were homogenous for most demographic and anamnestic parameters; only the proportion of patients that experienced diseases within three months prior to study start was significantly higher in the control group (see Table 2). Most patients had concomitant diseases and required concomitant medication.

**Table 2:** Baseline demographic and anamnestic parameters (ITT population)

Baseline Parameter	Test group (MCT/LCT/FO-derived n-3 PUFAs) *N* = 21	Control group (MCT/LCT) *N* = 21	*p*-value
Age (years, mean ± SD)	55.8 ± 15.1	58 ± 13.0	0.6160
Male / Female (%)	66.7 / 33.3	57.1 / 42.9	–
Weight (kg, mean ± SD)	62.7 ± 12.3	63.2 ± 10.1	0.9001
Height (cm, mean ± SD)	170.6 ± 9.9	174.1 ± 9.7	0.2523
BMI (kg/m^2^, mean ± SD)	21.4 ± 2.6	20.8 ± 2.3	0.4041
Diseases within last 3 months before study start (%)	76.2	100	0.0478
Concomitant diseases at study start (%)	85.7	100	0.2317
Concomitant medication at study start (%)	100	95.2	1.000
Oncological disease (%)	52.4	61.9	0.7557
Chemotherapy during last year (%)	14.3	23.8	0.6965
Radiation therapy during last year (%)	0	9.5	0.4878
Other tumor therapy during last year (%)	19.0	14.3	1.000
Weight loss during the last three months (%)	42.9	47.6	1.000
Nicotine consumption (%)	38.1	33.3	1.000
Alcohol abuse (%)	4.8	0	1.000
Drug abuse (%)	0	0	NA
On diet (not specified) before study start (%)	0	4.8	1.000

### Extent of exposure, treatment compliance

During the course of this study, the mean amount of lipid emulsion taken was 36.4 ± 11.4 bottles in the test group and 41.0 ± 10.4 bottles in the control group. As mean study duration was 48.0 ± 16.6 days and 59.1 ± 14.6 days in test and control group, respectively, this corresponded to a daily lipid intake of approximately 76 g in the test and 70 g in the control group. Patients in the test group therefore received about 7.6 g fractionated FO per day.

Treatment compliance, defined as ‘number of bottles of lipid emulsions used’/‘number of bottles of lipid emulsions prescribed’ was good and comparable between groups (90.9 and 94.8% in test and control groups, respectively).

### Efficacy of nutritional treatment

BMI increased in both groups after 8 weeks of HPN (ΔBMI_(test group)_ = 1.3 ± 1.1 kg/m^2^ and ΔBMI_(control group)_ = 0.6 ± 0.9 kg/m^2^) and analysis of treatment difference (mean difference ΔBMI(test group) – ΔBMI(control group) = 0.63 kg/m^2^) revealed that the lower margin of the 97.5% CI ([− 0.07; ∞]) exceeded the pre-defined inferiority level of − 1.1 kg/m^2^, indicating non-inferiority of the test lipid emulsion with respect to nutritional efficacy. BMI changes were neither correlated to the lipid emulsion assigned nor to the amount of lipid emulsion administered as revealed by covariance analyses.

BMI changes over the 8 weeks of HPN were based on a gain of body weight in both study groups (+ 3.7 ± 3.1 kg and + 2.0 ± 2.9 kg in test and control groups, respectively) which was also reflected by a comparable increase of body cell mass (BCM) in the test and control groups as determined via BIA (ΔBCM_(test group)_ = 3.4 ± 5.3%, ΔBCM_(control group)_ = 3.2 ± 7.7%). Covariance analyses revealed that weight gain was not correlated to the lipid emulsion assigned, the amount of lipid emulsion administered, weight loss within three month prior study start, or chemotherapy during the year before study start.

BMI increase and weight gain were more pronounced during the first 4 weeks of HPN: while body weight and BMI increased by 4.2 ± 3.9% and 2.6 ± 2.9% in test and control groups, respectively, during the first 4 weeks of HPN, BMI and body weight increased by 1.5 ± 3.0% and 0.6 ± 4.2% in test and control groups, respectively, during the subsequent 4 weeks of HPN (see also Table 3). Weight gain during the first 4 weeks of HPN was correlated to the conduct of chemotherapy during the year before study start.

**Table 3:** Changes of parameters for nutritional efficacy during HPN

Efficacy parameter	Test group (MCT/LCT/FO-derived n-3 PUFAs) *N* = 15	Control group (MCT/LCT) *N* = 18
BMI (kg/m^2^)		
Baseline	21.9 ± 2.4	20.7 ± 2.4
V1 (4 weeks HPN)	22.8 ± 2.2	21.2 ± 2.5
V2 (8 weeks HPN)	23.2 ± 2.6	21.3 ± 2.4
Mean treatment difference (V2-BL) = 0.63 kg/m^2^, CI 95%: [− 0.07; 1.32], *p* = 0.0768, t-test		
Body weight (kg)		
Baseline	63.1 ± 13.0	62.9 ± 10.7
V1 (4 weeks HPN)	65.6 ± 12.9	64.4 ± 10.8
V2 (8 weeks HPN)	66.7 ± 13.9	64.8 ± 11.0
Mean treatment difference (V2-BL) = 1.70 kg, CI 95%: [− 0.44; 3.84], *p* = 0.1153, t-test		
Body cell mass (kg)*		
Baseline	24.5 ± 6.3	22.9 ± 4.5
V1 (4 weeks HPN)	25.0 ± 6.0	23.7 ± 4.7
V2 (8 weeks HPN)	25.4 ± 6.7	24.4 ± 5.4
Mean treatment difference (V2-BL) = − 0.01 kg, CI 95%: [− 1.33; 1.32], *p* = 0.9939, t-test		

*Missing values (test group): N_miss_(Baseline, V1) = 1; N_miss_(V2) = 3 Missing values (control group): N_miss_(Baseline) = 2; N_miss_(V1, V2) = 1

### Influence of nutritional regimen on fatty acid pattern

Lipid composition of erythrocytes, platelets and serum phospholipids was significantly altered after 8 weeks of administration of the test lipid emulsion. In the test group, the proportion of n-3 PUFAs (i. e. Eicosapentaenoic acid (EPA), Docosahexaenoic acid (DHA) and Docosapentaenoic acid (DPA)) increased while the proportion of n-6 PUFAs (i.e. Linoleic Acid (LA), Arachidonic Acid (AA), Dihomo-ɣ-linolenic acid (DGLA) and ɣ-Linolenic acid (GLA)) decreased in erythrocytes, platelets and serum phospholipids. In the control group, the proportion of n-3 PUFAs and n-6 PUFAs remained mostly stable (see Table 4). Significant treatment differences were detected for EPA, DHA and DPA in erythrocytes, platelets and serum phospholipids. Significant treatment differences for n-6 PUFAs were found in erythrocytes (AA, DGLA and GLA), platelets (LA, DGLA and GLA) and serum phospholipids (LA, AA).

**Table 4:** Changes from Baseline of n-6 and n-3 PUFAs in Erythrocytes, Platelets and Serum Phospholipids upon 8 weeks of HPN

	Test group (MCT/LCT/FO-derived n-3 PUFAs) *N* = 11*	Control group (MCT/LCT) *N* = 9*
n-6 PUFAs		
Linoleic Acid (LA)		
*Erythrocytes (%)*		
Baseline	10.1 ± 1.0	10.2 ± 1.5
V2 (8 weeks HPN)	9.3 ± 0.9	10.6 ± 1.4
Mean treatment difference (V2-BL) = − 1.14%, CI 95 [− 2.0; − 0.3], *p* = 0.0107, t-test		
*Platelets (%)*		
Baseline	22.1 ± 3.1	16.9 ± 4.0
V2 (8 weeks HPN)	21.8 ± 2.9	20.9 ± 4.4
Mean treatment difference (V2-BL) = − 5.0%, CI 95 [− 7.3; − 2.7], *p* = 0.0002, t-test		
*Serum Phospholipids (%)*		
Baseline	20.0 ± 2.3	20.0 ± 3.1
V2 (8 weeks HPN)	18.1 ± 1.8	20.9 ± 2.6
Mean treatment difference (V2-BL) = − 3.3%, CI 95 [− 4.6; − 2.1, *p* < 0.0001, t-test		
Arachidonic Acid (AA)		
*Erythrocytes (%)*		
Baseline	16.8 ± 2.0	16.8 ± 1.3
V2 (8 weeks HPN)	13.2 ± 1.9	17.0 ± 2.4
Mean treatment difference (V2-BL) = − 3.8%, CI 95 [− 5.9; − 1.8], *p* = 0.0011, t-test		
*Platelets (%)*		
Baseline	10.9 ± 3.8	12.9 ± 4.0
V2 (8 weeks HPN)	8.4 ± 1.6	11.3 ± 4.2
Mean treatment difference (V2-BL) = − 1.3%, CI 95 [− 3.8; 1.2], *p* = 0.2883, t-test		
*Serum Phospholipids (%)*		
Baseline	10.6 ± 1.5	9.7 ± 2.2
V2 (8 weeks HPN)	8.2 ± 1.4	9.6 ± 1.9
Mean treatment difference (V2-BL) = − 2.7%, CI 95 [− 4.0; − 1.4], *p* = 0.0005, t-test		
Dihomo-γ-linolenic acid (DGLA)		
*Erythrocytes (%)*		
Baseline	2.2 ± 0.4	2.4 ± 0.5
V2 (8 weeks HPN)	1.7 ± 0.3	2.4 ± 0.5
Mean treatment difference (V2-BL) = − 0.4%, CI 95 [− 0.6; − 0.1], *p* = 0.0044, t-test		
*Platelets (%)*		
Baseline	1.7 ± 0.4	1.9 ± 0.4
V2 (8 weeks HPN)	1.1 ± 0.4	1.8 ± 0.4
Mean treatment difference (V2-BL) = − 0.4%, CI 95 [− 0.8; − 0.01], *p* = 0.0471, t-test		
*Serum Phospholipids (%)*		
Baseline	3.1 ± 1.0	4.2 ± 1.2
V2 (8 weeks HPN)	2.1 ± 0.9	3.8 ± 1.3
Mean treatment difference (V2-BL) = − 0.5%, CI 95 [− 1.2; 0.1], *p* = 0.1156, t-test		
γ-Linolenic acid (GLA)		
*Erythrocytes (%)*		
Baseline	0.08 ± 0.03	0.09 ± 0.4
V2 (8 weeks HPN)	0.05 ± 0.02	0.10 ± 0.05
Mean treatment difference (V2-BL) = − 0.05%, CI 95 [− 0.09; − 0.01], *p* = 0.0118, t-test		
*Platelets (%)*		
Baseline	0.5 ± 0.3	0.4 ± 0.1
V2 (8 weeks HPN)	0.3 ± 0.1	0.5 ± 0.2
Mean treatment difference (V2-BL) = − 0.3%, CI 95 [− 0.5; − 0.1], *p* = 0.0049, t-test		
*Serum Phospholipids (%)*		
Baseline	0.14 ± 0.08	0.16 ± 0.07
V2 (8 weeks HPN)	0.07 ± 0.04	0.13 ± 0.04
Mean treatment difference (V2-BL) = − 0.03%, CI 95 [− 0.09; 0.04], *p* = 0.3647, t-test		
n-3 PUFAs		
Eicosapentaenoic acid (EPA)		
*Erythrocytes (%)*		
Baseline	0.7 ± 0.2	0.7 ± 0.2
V2 (8 weeks HPN)	2.8 ± 0.9	0.7 ± 0.1
Mean treatment difference (V2-BL) = 2.1%, CI 95 [1.4; 2.8], *p* < 0.0001, t-test		
*Platelets (%)*		
Baseline	0.7 ± 0.2	0.6 ± 0.2
V2 (8 weeks HPN)	4.3 ± 2.0	0.6 ± 0.2
Mean treatment difference (V2-BL) = 3.4%, CI 95 [2.1; 4.8], *p* < 0.0001, t-test		
*Serum Phospholipids (%)*		
Baseline	1.1 ± 0.3	1.1 ± 0.4
V2 (8 weeks HPN)	4.6 ± 1.5	1.0 ± 0.3
Mean treatment difference (V2-BL) = 3.5%, CI 95 [2.5; 4.5], *p* < 0.0001, t-test		
Docosahexaenoic acid (DHA)		
*Erythrocytes (%)*		
Baseline	4.5 ± 1.3	4.1 ± 1.1
V2 (8 weeks HPN)	6.5 ± 1.1	4.0 ± 0.5
Mean treatment difference (V2-BL) = 2.2%, CI 95 [1.0; 3.3], *p* = 0.0008, t-test		
*Platelets (%)*		
Baseline	1.8 ± 0.4	1.4 ± 0.5
V2 (8 weeks HPN)	3.6 ± 0.7	1.5 ± 0.4
Mean treatment difference (V2-BL) = 1.8%, CI 95 [1.3; 2.3], *p* < 0.0001, t-test		
*Serum Phospholipids (%)*		
Baseline	3.1 ± 0.7	2.7 ± 1.0
V2 (8 weeks HPN)	5.8 ± 1.6	2.7 ± 0.6
Mean treatment difference (V2-BL) = 2.6%, CI 95 [1.6; 3.6], *p* < 0.0001, t-test		
Docosapentaenoic acid (DPA)		
*Erythrocytes (%)*		
Baseline	2.9 ± 0.5	2.9 ± 0.5
V2 (8 weeks HPN)	4.0 ± 0.5	3.0 ± 0.7
Mean treatment difference (V2-BL) = 0.8%, CI 95 [0.3; 1.4], *p* = 0.0058, t-test		
*Platelets (%)*		
Baseline	0.9 ± 0.2	1.1 ± 0.3
V2 (8 weeks HPN)	1.4 ± 0.2	1.0 ± 0.4
Mean treatment difference (V2-BL) = 0.5%, CI 95 [0.3; 0.7], *p* = 0.0003, t-test		
*Serum Phospholipids (%)*		
Baseline	1.0 ± 0.2	1.0 ± 0.3
V2 (8 weeks HPN)	1.6 ± 0.4	1.0 ± 0.3
Mean treatment difference (V2-BL) = 0.4%, CI 95 [0.2; 0.6], *p* = 0.0009, t-test		

* Number of baseline values (test group): *N* = 14; Number of baseline values (control group): *N* = 13

### Influence of nutritional regimen on inflammatory parameters

IL-10 and TNF-α values were within the reference range at baseline and stayed stable during nutritional treatment. IL-6 and CRP levels exceeded the reference range in both study groups at baseline, probably reflecting the high incidence of co-morbidities. Mean values of IL-6 and CRP increased in the test group during 8 weeks of HPN while they decreased in the control group. However, IL-6 and CRP values in the test group showed a broad distribution especially after 8 weeks of HPN (IL-6: min-value: 3.30 ng/l, max-value: 32.5 ng/l; CRP: min-value: 0.12 ng/l, max-value: 6.90 mg/dl) which is reflected by the high standard deviation for mean IL-6 and CRP-values in the test group (see Table 5). This indicates single outliers with a high impact on mean values due to the small number of patients included for this investigation (*N* = 11 in each group). No statistically significant differences could be detected between groups regarding the profile of inflammatory markers after 8 weeks of HPN Mean values of inflammatory parameters are displayed in Table 5.

**Table 5:** Inflammatory parameters before and after 8 weeks of HPN

Inflammatory parameter	Test group (MCT/LCT/n-3 PUFA) *N* = 11	Control group (MCT/LCT) *N* = 11
IL-6 (ng/L)		
(reference range < 5 ng/L)		
Baseline	5.473 ± 2.501	7.050 ± 5.432
V2 (8 weeks HPN)	9.145 ± 8.614	4.364 ± 2.448
Mean treatment difference (V2-BL) = 4.8 ng/L, CI 95 [− 0.5; 10.1], *p* = 0.0745, t-test		
IL-10 (ng/L)		
(reference range < 5 ng/L)		
Baseline	5.000 ± 0.000	5.033 ± 0.115
V2 (8 weeks HPN)	5.000 ± 0.000	5.027 ± 0.090
Mean treatment difference (V2-BL) = − 0.03 ng/L, CI 95 [− 0.09; 0.03], *p* = 0.3062, t-test		
TNF-alpha (ng/L)		
(reference range < 15 ng/L)		
Baseline	11.918 ± 6.506	13.175 ± 6.996
V2 (8 weeks HPN)	10.673 ± 4.382	10.645 ± 4.457
Mean treatment difference (V2-BL) = 0.9 ng/L, CI 95 [− 2.5; 4.3], *p* = 0.5801, t-test		
CRP (mg/dL)*		
(reference range < 0.50 mg/dL)		
Baseline	0.691 ± 0.595	0.977 ± 1.370
V2 (8 weeks HPN)	1.453 ± 1.903	0.745 ± 0.541
Mean treatment difference (V2-BL) = 0.6 mg/L, CI 95 [− 0.4; 1.6], *p* = 0.2231, t-test		

*Number of values (test group): N(Baseline) = 14; N(V2) = 15 Number of values (control group): N(Baseline) = 18; N(V2) = 18

### Influence of nutritional regimen on quality of life

Evaluation of the EORTC-QLQ-C30 questionnaire revealed that scores for global health status increased equally during treatment with the test and the control lipid emulsion (test group: 46.21 ± 12.56 (BL) vs 52.08 ± 20.14; control group: 36.27 ± 22.62 (BL) vs. 44.44 ± 24.73 (V2); score range: 0–100). Statistical analysis of score changes between BL and V2 revealed no significant treatment dependent differences.

### Safety of nutritional treatment

No differences could be detected between groups regarding the profile of laboratory parameters determined to monitor liver function, bone metabolism, renal function, metabolic activity, lipid metabolism, coagulation and haematology. All parameters stayed stable throughout the nutritional treatment (for mean values ± SD and reference ranges see Additional file 2). Only two individual clinically relevant abnormalities were reported (low platelet count, already present at baseline, and CRP elevation, reported as AE, both in the test group).

The number and intensity of reported adverse events (AEs) were comparable for test and control group. A total of 11 patients in the test group and 12 patients in the control group experienced at least one treatment emergent AE. In total, 76 AEs were reported (34 and 42 AEs in test and control groups, respectively).

No differences were detected regarding the AE pattern between study groups. Most AEs were classified as “Gastrointestinal disorders” (i.e. diarrhea, nausea and vomiting, constipation), “Musculoskeletal and connective tissue disorders” (mainly muscle spasm), “Nervous system disorders” (headache and somnolence), “General disorders and administration site conditions” (i.e. fatigue, chills and medical device complication), “Infections and infestations” (i.e. device related sepsis), and “Skin and subcutaneous tissue disorders”. None of the AEs was considered to be related to the nutritional regimen.

A total of four patients in each treatment group experienced at least one AE that was rated as serious. In total, 10 serious treatment emergent AEs were recorded. Although none of these serious adverse events (SAEs) was related to the investigational products, the treatment was prematurely terminated due to inability to continue IP administration during hospitalisation in six patients (*n* = 4 and *n* = 2 in test and control groups, respectively). No unexpected SAEs occurred. The most frequent SAE (device related sepsis) was expected as it represents a common complication of PN therapy. No patient died during the study.

## Discussion

HPN aims to prevent malnutrition in patients who cannot cover their nutritional requirements via the oral or enteral route for a prolonged period of time. This clinical trial was performed to compare the nutritional efficacy of two different lipid emulsions when administered as part of HPN for a duration of 8 weeks. Test and control lipid emulsions only differed in terms of lipid composition: the test lipid emulsion provided a mixture of MCT, LCT and FO-derived n-3 PUFAs (EPA and DHA) in a ratio of 5:4:1, while the control lipid emulsion provided a mixture of MCT and LCT in a 1:1 ratio.

Nutritional efficacy was assessed via changes of BMI during 8 weeks of HPN. BMI increased in both study groups during HPN treatment with a trend to higher BMI increases in the test group. Statistical analysis revealed non-inferiority of the test lipid emulsion to the reference lipid emulsion indicating that replacement of 10% LCT by FO-derived n-3 PUFAs does not affect nutritional efficacy and that PN regimes containing EPA and DHA are at least as efficient in maintaining as well as improving the nutritional status during HPN as PN regimens without those n-3 PUFAs.

It is well known that PUFAs are incorporated into cellular membranes [6]. In order to assess incorporation of PUFAs upon long-term HPN of 8 weeks, the FA pattern of cell-membrane phospholipids in erythrocytes and platelets as well as serum phospholipids was assessed in this clinical trial. FA analysis revealed significant treatment differences between test and control group. Upon administration of n-3 PUFA for a period of 8 weeks, the proportion of n-3 PUFAs (i. e. EPA, DHA and DPA) was increased while the proportion of n-6 PUFAs (AA, DGLA and GLA) was decreased in cell-membrane phospholipids in erythrocytes and platelets as well as in serum phospholipids. The FA pattern in the control group remained mostly stable. The effect of n-3 PUFAs administration during 8 weeks is thus in line with several other studies that investigated on the incorporation of PUFAs into serum and cell-membrane phospholipids after administration of lipid emulsions (reviewed in [30]).

The n-3/n-6 ratio of phospholipids in cell-membranes is thought to play an important role in the modulation of inflammation [10]. In response to an inflammatory stimulus AA (n-6 PUFA) and EPA (n-3 PUFA) are both released from cell membranes and are metabolized by the same enzymes into eicosanoids that modulate the intensity and duration of inflammatory responses [8]. An elevation of the n-3/n-6 ratio is thus thought to result in less intense inflammatory reactions and also reduced amounts of inflammatory cytokines that in turn might prevent the development of life-threatening hyper-inflammatory states. This assumption is supported by findings in gastrointestinal surgical patients that show beneficial modulation of eicosanoids and cytokines and reduced length of hospital stay after administration of FO as a source of bioactive n-3 PUFAs (reviewed in [20]). Beneficial effects of n-3 PUFA administration (reduction of infection rate, reduced length of intensive care unit (ICU) and hospital stay, increased release of less potent inflammatory mediators and reduction of inflammatory cytokines) have also been reported in a meta-analysis of a pooled population of surgical and medical ICU patients [31]. Nevertheless, the state of evidence is less clear in critically ill medical patients [11]. Meta-analyses of studies in critically ill patients (excluding studies with surgical intensive care patients) indicated that FO administration may reduce mortality and duration of ventilation [32] or reduce the incidence of infectious complications and length of hospital [33]. However, other studies did not reveal any beneficial effects of n-3 PUFA administration on cytokine levels or primary outcome parameters in this patient population [34, 35].

Although this clinical trial was not powered to address the influence of FO-derived n-3 PUFA administration on inflammatory parameters, serum cytokine levels were analysed for explorative purposes. IL-10 and TNF-α values were within the reference range at baseline and were not altered during 8 weeks of HPN. Most patients had elevated IL-6 and CRP values already at study start, likely reflecting the high incidence of concomitant diseases – nearly all patients that participated in this study suffered from concomitant diseases – and indicating an elevated inflammatory state in both groups at study start. There was a trend towards higher IL-6 and CRP levels in the group receiving n-3 PUFAs paralleled by a trend towards lower IL-6 and CRP levels in the control group. However, no statistically significant group differences were detected and time profiles determined for IL-6 and CRP levels have to be interpreted very carefully due to broad data distribution, reflecting individual abnormally high values, and small sample size. Results of this clinical trial therefore do not allow to reveal whether administration of n-3 PUFAs influence serum cytokine levels in HPN patients, and adequately powered studies are required to address this aspect.

Maintenance and improvement of nutritional status are especially important during HPN as malnutrition has been shown to decrease quality of life in patients with both benign and malignant diseases of the digestive system [36, 37]. Assessment of quality of life in this clinical trial revealed that scores for global health status and functional scales were increased after 8 weeks of HPN in both groups. This indicates increased quality of life and a better functioning in daily life that was most probably due to an improved nutritional status achieved via HPN therapy. This is in line with other studies showing an increase in quality of life due to HPN [38–40].

Coagulation parameters assessed in this study were similar between treatment groups, remained stable throughout 8 weeks HPN and were within the reference range. In addition, there were no adverse events indicative for an increased risk of bleeding. In line with other publications, data derived from this study therefore does not indicate an increased risk of bleeding upon administration of n-3 PUFAs [22, 23].

In this study, administration of both lipid emulsions was safe for a period of eight weeks. No treatment related AE (i.e. no adverse drug reaction) was reported and there was no difference in the occurrence of AEs or SAEs between the test and control groups. All safety laboratory parameters determined to monitor hepatic metabolism, haematology/coagulation, lipid metabolism, and bone metabolism remained stable during 8 weeks of HPN in both treatment groups. These findings are in line with other studies assessing safety of long-term administration of different lipid emulsions that revealed good clinical tolerance and safety of all lipid emulsions tested (reviewed in [30]).

One limitation of this study is that several study participants were on HPN therapy already at the study start. It was intended to include only patients with a new indication for HPN in this study. However, recruitment of patients was very difficult, as patients had to be able and willing to mix the HPN regimen at home. The inclusion criteria therefore had to be amended in order to also allow study participation of patients already receiving HPN. BMI changes detected during this study therefore most probably underestimate the beneficial effects of HPN on BMI. Furthermore, it cannot be excluded that differences between treatment groups (e.g. BMI increase, inflammatory parameters) would have been more pronounced if only patients with a new indication for HPN had been included. A further limitation is, that data regarding prior dietary intake was not collected. The correlation between prior HPN and treatment differences could therefore not be analysed. However, these limitations do not affect the assessment of efficacy and safety of the MCT/LCT/FO-derived n-3 PUFAs containing lipid emulsion.

## Conclusions

This study revealed that the lipid emulsion containing MCT/LCT/FO-derived n-3 PUFAs is at least as efficient in maintaining or improving the nutritional status of patients requiring long-term HPN as the lipid emulsion containing MCT/LCT only. Administration of FO-derived n-3 PUFAs for a period of 8 weeks markedly altered the fatty acid profile of serum and cell-membrane phospholipids resulting in an increased proportion of n-3 PUFAs and a decreased proportion of n-6 PUFAs. In this study, both lipid emulsions were safe and well tolerated during 8 weeks of HPN.

## Additional files

Additional file 1: CONSORT 2010 checklist of information to include when reporting a randomised trial, Provides the populated CONSORT 2010 checklist. (PDF 137 kb)

Additional file 2: Laboratory values and reference ranges. Displays mean values and standard deviation of laboratory data determined at Baseline, Visit 1 and Visit 2, used to assess the safety endpoints liver function, bone metabolism, renal function, metabolic activity, lipid metabolism, coagulation and haematology. (PDF 2040 kb)

## Abbreviations

(S)AE: (Serious) Adverse Event
BCM: Body Cell Mass
BIA: Bio Impedance Analysis
BL: Baseline Visit
BMI: Body Mass Index
CI: Confidence Interval
EN: Enteral Nutrition
FAS: Full Analysis Set
FO: Fish Oil
HPN: Home Parenteral Nutrition
ICU: Intensive Care Unit
ITT: Intent-to-Treat
LCT: Long-chain triglycerides
MCT: Medium-chain triglycerides
PN: Parenteral Nutrition
PP: Per Protocol
PUFA: Polyunsaturated Fatty Acids
SD: Standard Deviation
V1: Visit, 4 weeks after study start
V2: Visit, 8 weeks after study start

## References

[1] Staun M, Pironi L, Bozzetti F, Baxter J, Forbes A, Joly F (2009). ESPEN guidelines on parenteral nutrition: home parenteral nutrition (HPN) in adult patients. Clin Nutr.

[2] Pironi L, Goulet O, Buchman A, Messing B, Gabe S, Candusso M (2012). Outcome on home parenteral nutrition for benign intestinal failure: a review of the literature and benchmarking with the European prospective survey of ESPEN. Clin Nutr.

[3] McNamara MJ, Alexander HR, Norton JA (1992). Cytokines and their role in the pathophysiology of cancer cachexia. JPEN J Parenter Enteral Nutr.

[4] Bozzetti F, Gavazzi C, Ferrari P, Dworzak F (2000). Effect of total parenteral nutrition on the protein kinetics of patients with cancer cachexia. Tumori.

[5] Lawson RM, Doshi MK, Barton JR, Cobden I (2003). The effect of unselected post-operative nutritional supplementation on nutritional status and clinical outcome of orthopaedic patients. Clin Nutr.

[6] Wanten GJA, Calder PC (2007). Immune modulation by parenteral lipid emulsions. Am J Clin Nutr.

[7] Stapleton RD, Martin JM, Mayer K (2010). Fish oil in critical illness: mechanisms and clinical applications. Crit Care Clin.

[8] Calder PC (2015). Marine omega-3 fatty acids and inflammatory processes: effects, mechanisms and clinical relevance. Biochim Biophys Acta.

[9] Waitzberg DL, Torrinhas RS, Jacintho TM (2006). New parenteral lipid emulsions for clinical use. JPEN J Parenter Enteral Nutr.

[10] Miles EA, Calder PC (2015). Fatty acids, lipid emulsions and the immune and inflammatory systems. World Rev Nutr Diet.

[11] Calder PC, Adolph M, Deutz NE, Grau T, Innes JK, Klek S, et al. Lipids in the intensive care unit: recommendations from the ESPEN expert group. Clin Nutr. 2017. 10.1016/j.clnu.2017.08.032. 10.1016/j.clnu.2017.08.032 28935438

[12] Wachtler P, Konig W, Senkal M, Kemen M, Koller M (1997). Influence of a total parenteral nutrition enriched with omega-3 fatty acids on leukotriene synthesis of peripheral leukocytes and systemic cytokine levels in patients with major surgery. J Trauma.

[13] Grimm H, Mertes N, Goeters C, Schlotzer E, Mayer K, Grimminger F, Furst P (2006). Improved fatty acid and leukotriene pattern with a novel lipid emulsion in surgical patients. Eur J Nutr.

[14] Morlion BJ, Torwesten E, Lessire H, Sturm G, Peskar BM, Furst P, Puchstein C (1996). The effect of parenteral fish oil on leukocyte membrane fatty acid composition and leukotriene-synthesizing capacity in patients with postoperative trauma. Metabolism.

[15] Koller M, Senkal M, Kemen M, Konig W, Zumtobel V, Muhr G (2003). Impact of omega-3 fatty acid enriched TPN on leukotriene synthesis by leukocytes after major surgery. Clin Nutr.

[16] Wichmann MW, Thul P, Czarnetzki H-D, Morlion BJ, Kemen M, Jauch K-W (2007). Evaluation of clinical safety and beneficial effects of a fish oil containing lipid emulsion (Lipoplus, MLF541): data from a prospective, randomized, multicenter trial. Crit Care Med.

[17] Liang B, Wang S, Ye Y-J, Yang X-D, Wang Y-L, Qu J (2008). Impact of postoperative omega-3 fatty acid-supplemented parenteral nutrition on clinical outcomes and immunomodulations in colorectal cancer patients. World J Gastroenterol.

[18] Weiss G, Meyer F, Matthies B, Pross M, Koenig W, Lippert H (2002). Immunomodulation by perioperative administration of n-3 fatty acids. Br J Nutr.

[19] Jiang ZM, Wilmore DW, Wang XR, Wei JM, Zhang ZT, Gu ZY (2010). Randomized clinical trial of intravenous soybean oil alone versus soybean oil plus fish oil emulsion after gastrointestinal cancer surgery. Br J Surg.

[20] Calder PC (2013). Lipids for intravenous nutrition in hospitalised adult patients: a multiple choice of options. Proc Nutr Soc.

[21] Dyerberg J, Bang HO (1979). Haemostatic function and platelet polyunsaturated fatty acids in Eskimos. Lancet.

[22] Wachira JK, Larson MK, Harris WS (2014). N-3 fatty acids affect haemostasis but do not increase the risk of bleeding: clinical observations and mechanistic insights. Br J Nutr.

[23] Jeansen S, Witkamp RF, Garthoff JA, van Helvoort A, Calder PC (2018). Fish oil LC-PUFAs do not affect blood coagulation parameters and bleeding manifestations: analysis of 8 clinical studies with selected patient groups on omega-3-enriched medical nutrition. Clin Nutr.

[24] Jones CJ, Calder PC. Influence of different intravenous lipid emulsions on fatty acid status and laboratory and clinical outcomes in adult patients receiving home parenteral nutrition: a systematic review. Clin Nutr. 2016. 10.1016/j.clnu.2016.12.026. 10.1016/j.clnu.2016.12.026 28065480

[25] Klek S, Chambrier C, Singer P, Rubin M, Bowling T, Staun M (2013). Four-week parenteral nutrition using a third generation lipid emulsion (SMOFlipid)--a double-blind, randomised, multicentre study in adults. Clin Nutr.

[26] Driscoll DF, Ling P-R, Bistrian BR (2009). Pharmacopeial compliance of fish oil-containing parenteral lipid emulsion mixtures: globule size distribution (GSD) and fatty acid analyses. Int J Pharm.

[27] Versleijen MW, Roelofs HM, Rombouts C, Hermans PW, Noakes PS, Calder PC, Wanten GJ (2012). Short-term infusion of a fish oil-based lipid emulsion modulates fatty acid status, but not immune function or (anti)oxidant balance: a randomized cross-over study. Eur J Clin Investig.

[28] Barros KV, Cassulino AP, Schalch L, Della Valle Munhoz E, Manetta JA, Noakes PS (2013). Supplemental intravenous n-3 fatty acids and n-3 fatty acid status and outcome in critically ill elderly patients in the ICU receiving enteral nutrition. Clin Nutr.

[29] Friede T, Kieser M (2003). Blinded sample size reassessment in non-inferiority and equivalence trials. Stat Med.

[30] Pironi L, Agostini F, Guidetti M (2015). Intravenous lipids in home parenteral nutrition. World Rev Nutr Diet.

[31] Pradelli L, Mayer K, Muscaritoli M, Heller AR (2012). N-3 fatty acid-enriched parenteral nutrition regimens in elective surgical and ICU patients: a meta-analysis. Crit Care.

[32] Manzanares W, Dhaliwal R, Jurewitsch B, Stapleton RD, Jeejeebhoy KN, Heyland DK (2014). Parenteral fish oil lipid emulsions in the critically ill: a systematic review and meta-analysis. JPEN J Parenter Enteral Nutr.

[33] Manzanares W, Langlois PL, Dhaliwal R, Lemieux M, Heyland DK (2015). Intravenous fish oil lipid emulsions in critically ill patients: an updated systematic review and meta-analysis. Crit Care.

[34] Friesecke S, Lotze C, Köhler J, Heinrich A, Felix SB, Abel P (2008). Fish oil supplementation in the parenteral nutrition of critically ill medical patients: a randomised controlled trial. Intensive Care Med.

[35] Palmer AJ, Ho CKM, Ajibola O, Avenell A (2013). The role of ω-3 fatty acid supplemented parenteral nutrition in critical illness in adults: a systematic review and meta-analysis. Crit Care Med.

[36] Norman K, Kirchner H, Lochs H, Pirlich M (2006). Malnutrition affects quality of life in gastroenterology patients. World J Gastroenterol.

[37] Gupta D, Lis CG, Granick J, Grutsch JF, Vashi PG, Lammersfeld CA (2006). Malnutrition was associated with poor quality of life in colorectal cancer: a retrospective analysis. J Clin Epidemiol.

[38] Chambers A, Hennessy E, Powell-Tuck J (2006). Longitudinal trends in quality of life after starting home parenteral nutrition: a randomised controlled study of telemedicine. Clin Nutr.

[39] Culine S, Chambrier C, Tadmouri A, Senesse P, Seys P, Radji A (2014). Home parenteral nutrition improves quality of life and nutritional status in patients with cancer: a French observational multicentre study. Support Care Cancer.

[40] Vashi PG, Dahlk S, Popiel B, Lammersfeld CA, Ireton-Jones C, Gupta D (2014). A longitudinal study investigating quality of life and nutritional outcomes in advanced cancer patients receiving home parenteral nutrition. BMC Cancer.

