# Supplementary material for: Efficacy of a long-term home parenteral nutrition regimen containing fish oil-derived n-3 polyunsaturated fatty acids: a single-centre, randomized, double blind study
Source: Nutr J. 2018 Nov 30;17:113. doi: 10.1186/s12937-018-0419-x (PMC6271579; doi:10.1186/s12937-018-0419-x)
Supplement: Supplementary file 2 — Laboratory values and reference ranges. Displays mean values and standard deviation of laboratory data determined at Baseline, Visit 1 and Visit 2, used to assess the safety endpoints liver function, bone metabolism, renal function, metabolic activity, lipid metabolism, coagulation and haematology. (PDF 2040 kb) [file 12937_2018_419_MOESM2_ESM.pdf]

Additional File 2 - Laboratory values and reference ranges

Contents

1. Parameters of hepatic metabolism at Baseline, Visit 1 and Visit 2 2
2. Parameter for bone metabolism at Baseline, Visit 1 and Visit 2 3
3. Parameters of renal function at Baseline, Visit 1 and Visit 2 4
4. Metabolic markers at Baseline, Visit 1 and Visit 2 5
5. Parameters of lipid metabolism at Baseline, Visit 1 and Visit 2 7
6. Parameters of coagulation and hematology at Baseline, Visit 1 and Visit 2 8

1. Parameters of hepatic metabolism at Baseline, Visit 1 and Visit 2

Treatment group	Visit	N	N (miss)	MEAN	SD
Alanine Transaminase (ALT; U/l), reference range: < 34 in woman and < 45 in man					
Lipidem	Baseline	21	0	31.095	21.870
	Visit 1	15	0	30.200	19.005
	Visit 2	15	0	25.400	14.822
Lipofundin	Baseline	21	0	35.238	22.405
	Visit 1	19	1	41.263	31.873
	Visit 2	18	0	37.389	35.953
Aspartate Transaminase (AST; U/l), reference range: < 35 in woman and < 50 in man					
Lipidem	Baseline	21	0	29.619	13.193
	Visit 1	15	0	31.067	10.600
	Visit 2	15	0	28.600	11.685
Lipofundin	Baseline	21	0	30.476	17.915
	Visit 1	19	1	34.105	20.755
	Visit 2	18	0	34.278	30.932
ɤ- Glutamyl-Transferase (ɣ-GT; U/l), reference range: < 38 in woman and < 55 in man					
Lipidem	Baseline	21	0	128.381	173.591
	Visit 1	15	0	111.467	147.176
	Visit 2	15	0	113.800	185.639
Lipofundin	Baseline	21	0	87.667	112.880
	Visit 1	19	1	67.316	73.893
	Visit 2	18	0	61.222	75.491
Alkaline Phosphatase (AP; U/l), reference range: 35 - 104 in woman and 40 - 129 in man					
Lipidem	Baseline	21	0	109.238	79.735
	Visit 1	15	0	114.133	83.796
	Visit 2	15	0	110.533	88.147
Lipofundin	Baseline	21	0	106.143	73.285
	Visit 1	18	2	101.889	62.640
	Visit 2	17	1	91.412	54.644
Bilirubin (mg(dl), reference range: < 1.0 mg/dl					
Lipidem	Baseline	21	0	0.517	0.325
	Visit 1	14	1	0.604	0.346
	Visit 2	15	0	0.617	0.444
Lipofundin	Baseline	21	0	0.413	0.194
	Visit 1	19	1	0.433	0.230
	Visit 2	18	0	0.384	0.153

2. Parameter for bone metabolism at Baseline, Visit 1 and Visit 2

Treatment group	Visit	N	N (miss)	MEAN	SD
Ostase (ug/l), reference range: 6.5 – 22.4 ug/l in woman (post menopause) and 6.0 – 20.1 ug/l in man					
Lipidem	Baseline	16	1	16.681	11.172
	Visit 1	11	0	18.273	13.177
	Visit 2	10	2	16.780	15.583
Lipofundin	Baseline	12	4	18.925	12.837
	Visit 1	12	4	12.208	5.433
	Visit 2	11	3	15.064	9.898

3. Parameters of renal function at Baseline, Visit 1 and Visit 2

Treatment group	Visit	N	N (miss)	MEAN	SD
Creatinine (mg/dl), reference range: < 1.0 mg/dl in woman and < 1.2 mg/dl in man					
Lipidem	Baseline	21	0	0.819	0.337
	Visit 1	15	0	0.940	0.447
	Visit 2	15	0	0.950	0.487
Lipofundin	Baseline	21	0	0.833	0.382
	Visit 1	19	1	0.845	0.464
	Visit 2	18	0	1.003	0.764
Urea (mg/dl), normal range: 14 - 46 mg/dl					
Lipidem	Baseline	21	0	38.905	17.401
	Visit 1	15	0	38.133	15.113
	Visit 2	15	0	34.800	13.224
Lipofundin	Baseline	21	0	40.619	23.794
	Visit 1	19	1	40.632	19.062
	Visit 2	18	0	45.056	26.651

4. Metabolic markers at Baseline, Visit 1 and Visit 2

Treatment group	Visit	N	N (miss)	MEAN	SD
Albumin (g/dl), reference range: 3.6 – 5 g/dl					
Lipidem	Baseline	21	0	5.727	7.698
	Visit 1	15	0	4.037	0.495
	Visit 2	15	0	6.473	9.339
Lipofundin	Baseline	21	0	3.929	0.477
	Visit 1	19	1	4.042	0.337
	Visit 2	18	0	7.903	11.598
Lactate (mg/dl), reference range: 6 – 18 mg/dl					
Lipidem	Baseline	21	0	13.814	4.700
	Visit 1	14	1	12.064	4.412
	Visit 2	13	2	12.285	3.873
Lipofundin	Baseline	21	0	15.886	6.016
	Visit 1	19	1	14.242	5.278
	Visit 2	18	0	13.878	4.481
Glucose (mg/dl), reference range: 55 – 110 mg/dl					
Lipidem	Baseline	21	0	107.619	42.642
	Visit 1	14	1	104.214	36.491
	Visit 2	14	1	96.000	49.917
Lipofundin	Baseline	20	1	102.650	42.022
	Visit 1	19	1	94.789	22.763
	Visit 2	18	0	98.444	52.653
pH, reference range: 7.34 – 7.44					
Lipidem	Baseline	20	1	7.380	0.032
	Visit 1	15	0	7.375	0.044
	Visit 2	15	0	7.373	0.033
Lipofundin	Baseline	20	1	7.355	0.058
	Visit 1	20	0	7.358	0.048
	Visit 2	18	0	7.369	0.063
Sodium (mmol/l), reference range: 134 – 145 mmol/l					
Lipidem	Baseline	21	0	138.619	2.519
	Visit 1	15	0	138.800	2.933
	Visit 2	15	0	139.467	3.159
Lipofundin	Baseline	21	0	138.952	3.667
	Visit 1	19	1	139.053	2.838
	Visit 2	18	0	138.500	3.869

Additional File 2 - Laboratory values and reference ranges

Treatment group	Visit	N	N (miss)	MEAN	SD
Potassium (mmol/l), reference range: 3.4 – 5.2 mmol/l					
Lipidem	Baseline	21	0	3.900	0.500
	Visit 1	15	0	3.920	0.378
	Visit 2	15	0	3.947	0.498
Lipofundin	Baseline	21	0	3.900	0.489
	Visit 1	19	1	3.821	0.437
	Visit 2	18	0	3.783	0.499
Calcium (mmol/l), reference range: 2.15 – 2.65 mmol/l					
Lipidem	Baseline	21	0	2.240	0.153
	Visit 1	15	0	2.230	0.192
	Visit 2	15	0	2.215	0.183
Lipofundin	Baseline	21	0	2.256	0.136
	Visit 1	19	1	2.268	0.128
	Visit 2	18	0	2.258	0.139
Magnesium (mmol/l), reference range: 0.75 – 1.06 mmol/l					
Lipidem	Baseline	21	0	0.765	0.085
	Visit 1	15	0	0.812	0.088
	Visit 2	15	0	0.819	0.079
Lipofundin	Baseline	21	0	0.810	0.084
	Visit 1	19	1	0.844	0.089
	Visit 2	17	1	0.848	0.181
Chloride (mmol/l), reference range: 95 – 112 mmol/l					
Lipidem	Baseline	21	0	102.143	3.651
	Visit 1	15	0	102.600	3.924
	Visit 2	15	0	103.333	3.266
Lipofundin	Baseline	21	0	102.619	6.281
	Visit 1	19	1	102.684	5.935
	Visit 2	18	0	101.444	6.128

5. Parameters of lipid metabolism at Baseline, Visit 1 and Visit 2

Treatment group	Visit	N	N (miss)	MEAN	SD
Triglycerides (mg/dl), reference range: < 150 mg/dl					
Lipidem	Baseline	21	0	105.190	35.966
	Visit 1	15	0	105.933	37.429
	Visit 2	15	0	107.067	51.587
Lipofundin	Baseline	21	0	135.429	73.165
	Visit 1	19	1	144.684	108.546
	Visit 2	18	0	126.278	65.135
Total cholesterol (mg/dl). Reference range: < 200 mg/dl					
Lipidem	Baseline	21	0	166.190	35.132
	Visit 1	15	0	181.067	34.077
	Visit 2	15	0	190.267	41.441
Lipofundin	Baseline	21	0	163.619	38.547
	Visit 1	19	1	178.000	45.749
	Visit 2	18	0	179.611	46.496
HDL cholesterol (mg/dl), reference range: > 45 mg/dl in woman and > 35 mg/dl in man					
Lipidem	Baseline	18	3	46.444	9.382
	Visit 1	12	3	49.667	16.422
	Visit 2	15	0	49.400	14.549
Lipofundin	Baseline	19	2	41.105	12.613
	Visit 1	17	3	42.824	12.700
	Visit 2	16	2	41.438	8.516
LDL cholesterol (mg/dl), reference range, < 130 mg/dl					
Lipidem	Baseline	18	3	88.667	25.564
	Visit 1	12	3	114.583	21.377
	Visit 2	15	0	110.133	36.528
Lipofundin	Baseline	19	2	86.053	25.971
	Visit 1	17	3	97.471	29.285
	Visit 2	16	2	93.375	30.960
Vitamin E(µmol/l), reference range: 12 – 46 µmol/l					
Lipidem	Baseline	18	3	28.200	6.117
	Visit 1	14	1	38.879	11.495
	Visit 2	15	0	40.293	12.756
Lipofundin	Baseline	21	0	26.438	9.419
	Visit 1	20	0	38.890	12.787
	Visit 2	17	1	41.182	14.327

6. Parameters of coagulation and hematology at Baseline, Visit 1 and Visit 2

Treatment group	Visit	N	N (miss)	MEAN	SD
aPTT (s), reference range: 26 – 38 s					
Lipidem	Baseline	21	0	35.419	4.124
	Visit 1	15	0	35.113	4.466
	Visit 2	15	0	34.727	3.215
Lipofundin	Baseline	21	0	36.962	4.178
	Visit 1	20	0	36.115	4.204
	Visit 2	18	0	39.550	18.462
PT (%), reference range: 70 – 130 %					
Lipidem	Baseline	21	0	87.857	14.623
	Visit 1	15	0	91.200	8.274
	Visit 2	15	0	89.333	6.904
Lipofundin	Baseline	21	0	93.714	11.226
	Visit 1	20	0	95.050	13.089
	Visit 2	18	0	93.322	18.172
Platelets (/nl), reference range: 150 – 400 /nl					
Lipidem	Baseline	21	0	218.714	76.447
	Visit 1	15	0	202.267	87.259
	Visit 2	15	0	206.133	76.452
Lipofundin	Baseline	21	0	305.095	121.432
	Visit 1	20	0	247.250	95.569
	Visit 2	18	0	245.944	118.025
Leukocytes (/nl), reference range: 4.5 – 11.0 /nl					
Lipidem	Baseline	21	0	6.932	2.257
	Visit 1	15	0	6.547	2.982
	Visit 2	15	0	6.911	2.206
Lipofundin	Baseline	21	0	6.740	2.076
	Visit 1	20	0	6.002	2.046
	Visit 2	18	0	6.389	2.306
Erythrocytes (/pl), reference range: 3.9 - 5.4 in woman and 4.6 – 6.2 in man					
Lipidem	Baseline	21	0	4.160	0.446
	Visit 1	15	0	4.269	0.494
	Visit 2	15	0	4.163	0.499
Lipofundin	Baseline	21	0	4.004	0.394
	Visit 1	20	0	3.991	0.519
	Visit 2	18	0	4.004	0.469

Additional File 2 - Laboratory values and reference ranges

Treatment group	Visit	N	N (miss)	MEAN	SD
Hematocrit (l/l), reference range: 0.35 – 0.47 l/l in woman and 0.40 – 0.52 l/l in man					
Lipidem	Baseline	21	0	0.376	0.037
	Visit 1	15	0	0.383	0.044
	Visit 2	15	0	0.374	0.040
Lipofundin	Baseline	21	0	0.356	0.039
	Visit 1	20	0	0.354	0.043
	Visit 2	18	0	0.357	0.040
Haemoglobin (g/dl), reference range: 12 – 15.7 g/dl in woman an 14 – 17.5 g/dl in man					
Lipidem	Baseline	21	0	12.552	1.301
	Visit 1	15	0	12.787	1.686
	Visit 2	15	0	12.487	1.670
Lipofundin	Baseline	21	0	11.548	1.535
	Visit 1	20	0	11.675	1.500
	Visit 2	18	0	11.722	1.313
Transferrin (mg/dl), reference range: 200 – 360 mg/dl					
Lipidem	Baseline	20	1	232.215	48.672
	Visit 1	15	0	263.067	60.278
	Visit 2	15	0	248.000	44.742
Lipofundin	Baseline	21	0	253.952	52.098
	Visit 1	19	1	257.158	58.114
	Visit 2	16	2	239.875	56.618
